# Exploring Selenide Synthesis Pathways for Optimizing Energy Conversion

**DOI:** 10.3390/molecules29143310

**Published:** 2024-07-13

**Authors:** Anna Kusior, Fabian Wieczorek, Jakub Dechnik, Andrzej Mikuła

**Affiliations:** 1 Faculty of Materials Science and Ceramics, AGH University of Krakow, al. Mickiewicza 30, 30-059 Kraków, Poland; jakubdechnik@student.agh.edu.pl (J.D.); amikula@agh.edu.pl (A.M.); 2 Faculty of Electrical Engineering, Automatics, Computer Science and Biomedical Engineering, AGH University of Krakow, al. Mickiewicza 30, 30-059 Kraków, Poland; fwieczorek@student.agh.edu.pl

**Keywords:** multicomponent system, transition metal selenides, electrochemical activity, Raman spectroscopy, solvothermal synthesis

## Abstract

This study investigated the structural and electrochemical characteristics of binary and quaternary systems comprising nickel, cobalt, and iron selenides. The powders were obtained via a solvothermal route. X-ray diffraction (XRD) and Raman spectroscopy revealed significant phase diversity. It was observed that increasing the proportion of d-block metals in quaternary systems enhances structural entropy, potentially leading to more homogeneous and stable structures dominated by energetically preferred components such as nickel. The electrochemical analysis indicated that the binary system exhibited a reversible redox reaction, with nickel selenide-based samples demonstrating the highest electrochemically active surface area. Quaternary systems display varying degrees of electrochemical stability. An equal contribution of nickel, cobalt, and iron appears beneficial in achieving stable electrodes. This research contributes to understanding the relationship between transition metal selenides’ structural, morphological, and electrochemical properties, providing insights into their potential applications in hydrogen generation.

## 1. Introduction

The hydrogen evolution reaction (HER) is considered an efficient route for hydrogen (H_2_) generation without pollution [1,2]. To date, due to the superior electrocatalytic activity, stability, and high cathodic current densities at low potentials, platinum has been considered the most effective catalyst for the HER [3,4,5]. Nevertheless, it is an expensive and rare material. Therefore, enormous efforts have been devoted to developing efficient alternatives, and remarkable progress has been achieved.

Across various materials, researchers are focusing on cost-effective transition metal dichalcogenides, such as MoS_2_ [6], CoS_2_ [7], FeSe_2_ [8], and NiSe_2_ [9]. These materials exhibit distinguished HER performance and good catalytic stability. However, compared to the noble catalyst, there remains a significant gap in the onset potential and current density. One of the potential solutions to enhance electrocatalytic activity is the incorporation or mixing of metals, which can influence electronic effects and thereby improve catalytic activity [2,10,11]. The ability of these materials to crystallize in simple cubic structures under large lattice strain, due to the different sizes of the constituent atoms, facilitates a two-stage hydrogen absorption reaction. This reaction begins with an intermediate monohydride and eventually forms a dihydride phase [12], resulting in a higher bulk density of hydrogen. Recently, researchers have focused on developing binary, ternary, and quaternary transition metal chalcogenides due to their partially filled d-orbitals, which mediate the gain and loss of electrons [13,14,15,16]. 

However, a systematic investigation of the catalytic activity trend as a function of transition metal solid-solution selenides is rare. Various processes have been employed to synthesize these materials, including selenization [17], solid-state reaction [14], mechanical alloying [18], high-temperature and high-pressure techniques [19], and metal–organic chemical vapor deposition (Table 1). Low-temperature synthetic routes, such as hydrothermal and solvothermal techniques, are also promising [20,21]. These methods do not require high-purity single compounds, reduce the complexity of synthesis, and allow for the production of particles with controllable shapes [22,23,24].

This study presents the structural and electrochemical investigations of the binary and quaternary systems of the transition metal selenides synthesized via the solvothermal route. The search for alternative methods of obtaining multicomponent materials aims to utilize cheaper and more readily available raw materials. This research focuses on determining the parameters of solvothermal synthesis conditions that, without using pure elements, would allow us to obtain an efficient amount of material while maintaining optimal properties.

## 2. Results and Discussion

**Figure 1:**
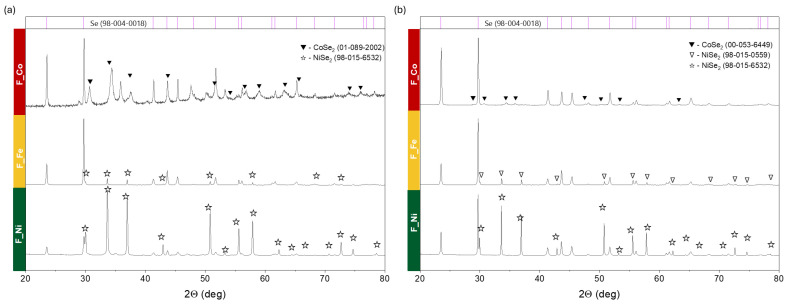
The XRD data of the obtained nickel, iron, and cobalt selenide samples after 24 (**a**) and 72 h (**b**) reaction at 180 °C.

**Figure 2:**
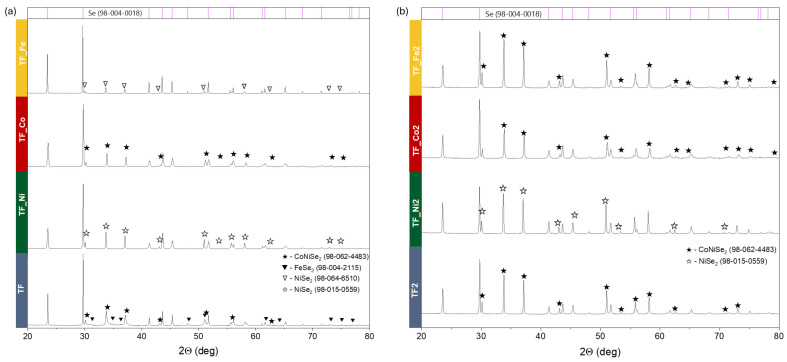
The XRD data of the obtained quaternary system transition metal selenide samples after 24 (**a**) and 72 h (**b**) reactions at 180 °C.

Figure 1 and Figure 2 present the X-ray diffraction (XRD) patterns for the selenide binary systems synthesized over 24 h and 72 h, respectively.

Regardless of the reaction time, the iron- and nickel-based powders crystallize in the cubic structure characteristic of NiSe_2_ (Pnnm). Broad reflections typical for fine crystallites are observed in the cobalt selenides. After 24 h, the structure is predominantly cubic CoSe_2_ (PDF #01-089-2002). However, the composition of F_Co2 is different, comprising an orthorhombic form (PDF #00-053-6449).

Despite the reaction time, a large amount of selenium with a hexagonal structure (PDF #98-002-2251) is visible in all samples. This indicates that the reaction did not proceed fully according to the stoichiometric assumptions. Specifically, the amount of metal used was insufficient to completely react with the selenide oxide, resulting in an excess of selenium in its metallic form. No other characteristic reflections were detected. However, extending the reaction time impacts the crystallinity level of the powders.

**Figure 3:**
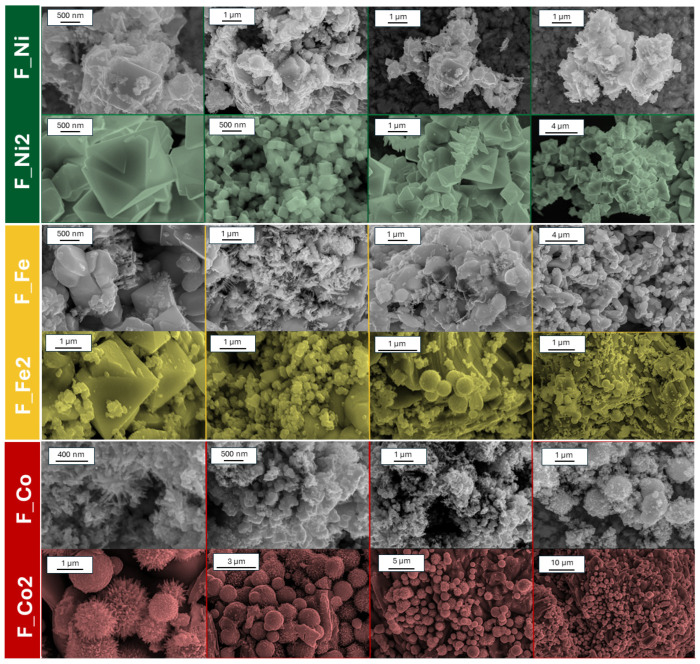
The SEM images of the obtained nickel, iron, and cobalt selenide samples after 24 and 72 h reaction at 180 °C.

Quaternary system materials were synthesized, assuming that introducing additional d-block metals would translate into a system with higher configurational entropy. The phase composition of the materials obtained is shown in Figure 3.

Similar to the binary system, the obtained materials are characterized by the presence of metallic selenium (PDF #98-004-0018), with the quantity varying depending on the composition and synthesis parameters. After a 24 h solvothermal process (Figure 2a), where the molar ratio of Ni:Co:Fe was 1:1:1 (sample TF), cubic CoNiSe_4_ (PDF #98-062-4483) and the orthorhombic-phase FeSe_2_ (PDF #98-004-2115) were detected. The second phase disappears with an increase in reaction time (Figure 2b). The composition changes from 40% Se, 40% CoNiSe_4_, and 20% FeSe_2_ to 50%/50% composition (TF2).

Increasing the amount of one component relative to the others significantly affects the resulting structure. For nickel-rich materials, a cubic system of the NiSe_2_ structure (PDF #98-015-0559) was obtained. However, 70% of the phase composition was contributed to selenium. Extending the process duration results in a higher yield of the desired material, reaching up to 55% (sample TF_Ni2). A similar trend was observed for cobalt- and iron-rich systems. In the first case, 25% of the material exhibited a CoNiSe_2_ structure (PDF #98-062-4482) after a 24 h process, while in the iron-rich system, 20% of the material had a cubic NiSe_2_ (PDF #98-064-6510) form. After 72 h of reaction, both samples were characterized by the presence of the cubic form of CoNiSe_2_ in a ratio of 45%/55% relative to selenium (Figure 2b).

**Figure 4:**
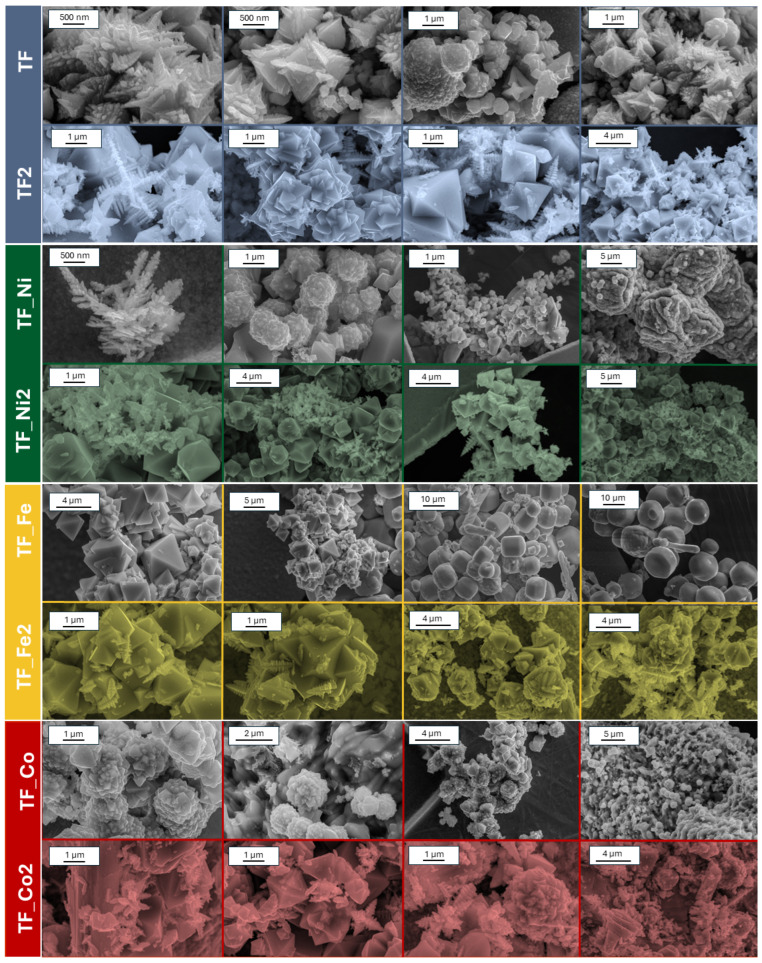
The SEM images of the obtained quaternary system transition metal selenide samples after 24 and 72 h reaction at 180 °C.

Scanning electron microscopy (SEM) measurements were performed to identify the micromorphology features of the prepared samples. Figure 3 and Figure 4 show the synthesized materials. All powders of the binary system exhibit a dual structure (Figure 3). The larger polyhedral grains observed in each material can be attributed to selenium. Nickel selenide is characterized by particles with sharp edges, while finer oval/spherical ones are observed for iron selenides. Notably, the F_Co and F_Co2 samples exhibit urchin-like forms. The powder morphology appears to be unaffected by the reaction parameters. However, a longer reaction time results in a coarse grain size. It may be assumed that, due to the Ostwald ripening mechanism, the smaller grains dissolve and recrystallize on the surface of the larger grains.

Interestingly, the variety of forms observed in binary systems, depending on the cation, is not reflected in quaternary systems (Figure 4). While after the 24 h reaction, the obtained structures may slightly resemble those of binary systems, after 72 h, it appears that the nickel-based structure begins to dominate. Consequently, the powders obtained are characterized by polyhedral grains with sharp edges.

Furthermore, the lack of variety in quaternary systems implies that the complexity introduced by additional components might diminish the influence of individual cations on the overall structure. This implies that the interactions among multiple elements in quaternary systems lead to a more homogenized or stabilized structure, predominantly governed by the energetically favorable component, such as nickel.

The formation of polyhedral grains with sharp edges in nickel-based quaternary structures could significantly impact the material’s properties, including surface area, reactivity, and mechanical strength. The varying reaction times and compositions are crucial to tailoring the material for specific applications, such as catalysis, electronics, or structural components.

**Figure 5:**
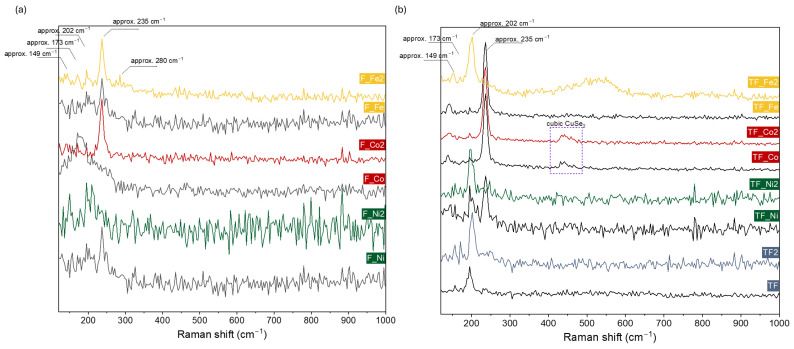
The Raman spectra of the obtained nickel, iron, and cobalt selenide (**a**) and the quaternary system transition metal selenide samples (**b**).

Raman scattering is considered a highly sensitive technique, particularly in surface analysis. However, in the case of selenide-based materials, a Raman analysis of pure samples has not been extensively pursued due to the lack of interrelated knowledge. Based on the available reports of the investigation, the characteristic vibration bands at approximately 149 (T_g_), 202 (A_g_) cm^−1^ and 173 (E_g_), 235 (T_g_) cm^−1^ can be assigned to the stretching and librational modes of the Se-Se bonds (Figure 5a,b), respectively [9,30]. The active mode at 280 cm^−1^ can be attributed to the B_1g_ Se-Se stretching mode, which is visible in iron-based selenides [20]. Notably, in monometallic selenides, strong vibrations are more visible for samples received after a longer synthesis time, particularly those containing cobalt and iron elements. Furthermore, the characteristic of the cubic Co-Se vibration mode at 468 cm^−1^ (Figure 5b) is evident [18].

An important observation is the relationship between the bands 202 and 235 cm^−1^, especially noticeable for quaternary systems (Figure 5b). For powders initially assumed to have an equal molar ratio of metals but be rich in nickel, the band associated with stretching vibrations dominates. Conversely, for samples rich in cobalt, the band 235 cm^−1^ related to the reciprocating motion is the most intensive. This suggests that cobalt during the crystallization process assumes specific privileged positions, forming a separate sublattice in which iron and nickel ions may be randomly placed. However, the presence of Ni influences the formation of the ordered structure, as evidenced by the TF_Fe and TF_Fe2 materials. With prolonged synthesis times and crystallization, the dominance of the bonds changes.

**Figure 6:**
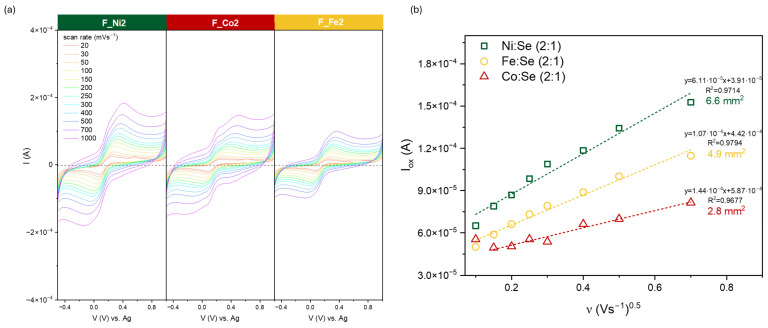
Current–voltage dependence (**a**) and the plot of the anodic peak potentials as a function of the square root of the scan rate (**b**) of nickel, cobalt, and iron selenides in 0.1 M KCl + 3 mM [Fe(CN)_6_]^3−/4−^.

**Figure 7:**
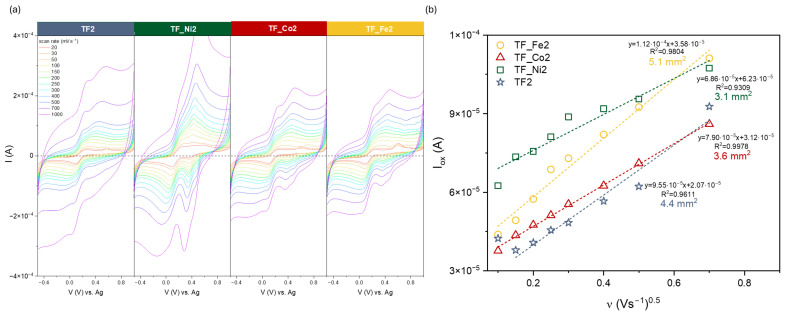
Current–voltage dependence (**a**) and the plot of the anodic peak potentials as a function of the square root of the scan rate (**b**) of the quaternary in 0.1 M KCl + 3 mM [Fe(CN)_6_]^3−/4−^.

The materials were investigated as supported electrodes to estimate the potential electrochemical activity and potential performance toward hydrogen evolution. The obtained powders were applied to the screen-printed electrodes using the drop-casting technique. The cyclic voltammetry recorded in the ferrocyanide/ferricyanide solution (0.1 M KCl + 3 mM [Fe(CN)_6_]^3−/4−^) was used for the evaluation of the electrochemically active surface area (EASA) and is presented in Figure 6 and Figure 7. Analyses were performed for samples obtained after a 72 h solvothermal reaction.

Binary systems (Figure 6a) are characterized by two oxidation peaks. The second signal becomes apparent as the scanning rate increases, indicating the presence of sites with different catalytic activity. The redox reactions are fully reversible and based on electron transfer. The ratio of the maximum peaks at the anodic (i_a_) and cathodic (i_c_) current parts is close to 1 (F_Ni2 = 1.01, F_Co2 = 0.97, and F_Fe2 = 0.97). Additionally, the observed curve types suggest perpendicular and radial diffusion mass transport.

Based on the results, the Randles–Sevcik equation was used to determine the electrochemically active surface area (EASA):

I = 2.69·10^5^·EASA·Dn^3/2^·C_0_·ν^1/2^ (1)

where I is the peak current (A); D—diffusion coefficient of [Fe(CN)_6_]^3−/4−^, 7.2·10^−6^ cm^2^s^−1^; n—number of electrons involved in the redox reaction (n = 1); C_0_—concentration of redox species (3 mM); and ν—scan rate (mVs^−1^). Analysis indicates that the highest EASA of 6.6 mm^2^ possessed the nickel selenide-based sample (Figure 6b).

Figure 7a shows the electrochemical behavior of the materials of the quaternary system. Each current–voltage curve exhibits two peaks. The positions of the first one (at lower potential values) are characteristic of the reaction of the material with [Fe(CN)_6_]^3−/4−^. The second signal possibly results from the interaction of the dominant cation in the system with the electrolyte. However, unlike binary systems, not all redox reactions observed are reversible, impacting the number of catalytically active sites and thus the efficiency of the material and its chemical stability under given conditions. This issue primarily affects the nickel- and iron-rich system (samples TF_Ni2 and TF_Fe2). TF2 appears to be the most promising material among the samples, with an EASA size of about 4.4 mm^2^ (Table 2). Since the samples are not entirely composed of pure selenides, further efforts are needed to refine the synthesis conditions. Moreover, preliminary electrochemical analyses suggest that assuming equal contributions of nickel, cobalt, iron (TF2), and the additional amount of nickel (TF_Ni2) results in a chemically stable electrode. The calculated ratio of the redox reaction maximum peak is 0.97 and 1.00, respectively. On the other hand, for samples rich in cobalt (TF_Co2) and iron (TF_Fe2), the quasi-reversible process occurs, and i_a_/i_c_ is 0.93 and 0.71, respectively. This may suggest that, except for electron exchange, the chemical reaction takes place.

**Figure 8:**
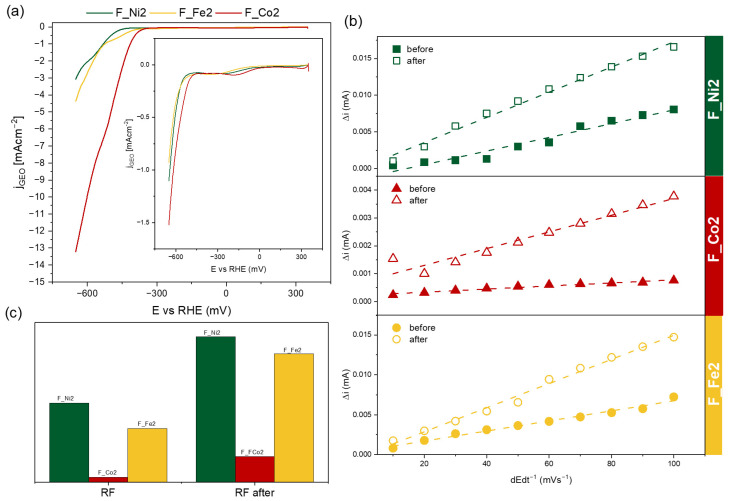
(**a**) LSV curves normalized to the geometrical surface area before and after measurements (inset), (**b**) changing currents as a function of the scan rate representing C_DL_, and (**c**) RF before and after the measurements of the electrodes.

**Figure 9:**
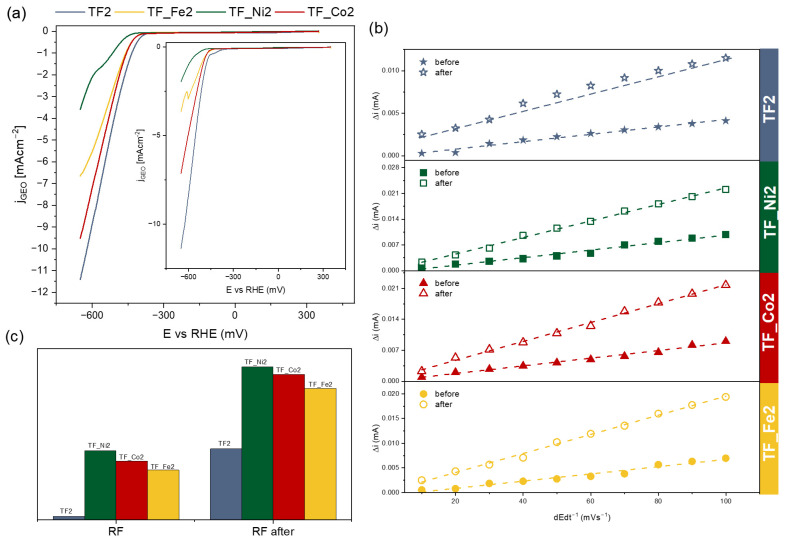
(**a**) LSV curves normalized to the geometrical surface area before and after measurements (inset), (**b**) changing currents as a function of the scan rate representing C_DL_, and (**c**) RF before and after the measurements of the electrodes.

HER activity was investigated based on LSV and CV measurements recorded in 0.5 M H_2_SO_4_. The LSV curves normalized to the geometrical surface area before and after stability tests, changes in current as a function of the scan rate, and roughness factor (RF) are shown in Figure 8 and Figure 9 for binary and quaternary systems, respectively.

The recorded current values for all samples are low due to the screen-printed electrodes’ low and rather uncertain coverage (Figure 8a). This phenomenon is caused by the co-occurrence of metal-like particles (MSe_2_) and free Se in the studied materials, significantly affecting the physisorption of powders on the electrode surfaces. Typically, the benchmark value for comparing HER activity is the overpotential at −10 mAcm^−2^ (η_10_), but only the F_Co2 sample achieves this value. Therefore, the comparative value adopted −2 mAcm^−2^ (η_2_). The lowest η_2_ vs. RHE is reached by Co-rich samples. Similarly, the slope of the LSV curve suggests the fastest reaction kinetics. At the same time, based on the current curves (Figure 8b), samples rich in Co show the lowest ECSA represented by the double-layer capacitance (CDL) and thus the lowest roughness factor (RF, Figure 8c), suggesting the lowest number of active sites. This seemingly contradictory result may be related to two aspects: the high Se content blocking the surface of CoSe_2_ (limited active sites) and the high affinity of Co-based active sites for hydrogen (highest activity determined by η_2_). In particular, this seems reasonable given the relatively small amount of CoSe_2_ phase compared to Se, as suggested by the intensity ratios of the reflections in the XRD. On the other hand, CoSe_2_ is present in a poorly ordered orthorhombic structure (further supported by Raman study), which may exhibit better activity towards HER. Despite the low overpotential, the highest number of active sites was offered by the F_Ni2 material (Figure 8c). This also seems reasonable because this sample is rich in the cubic phase, which provides greater accessibility to active sites not covered by Se (based on the intensity ratio in XRD). Meanwhile, materials with Ni are generally the least active towards HER [31]. All materials show increased activity and ECSA after measurements. This could be related to the formation of defects in the anionic sublattice, revealing metallic, active sites (electrochemical activation typical for some chalcogenide-based catalysts [32]), as well as the detachment of Se particles, which increases the availability of an actual catalyst for contact with the electrolyte. This is undoubtedly an issue that requires further investigation. However, to make these studies reliable, a further optimization of synthesis conditions to eliminate excess Se is necessary. The results are summarized in Table 2.

The results for quaternary systems (Figure 9) follow the trends observed for binary selenides. Although the recorded current densities are still low, they are noticeably higher than those for binary systems. The increased amount of cobalt indicates the best activity, confirming that Co-based active sites in these selenides exhibit a high affinity for HER. At the same time, Ni-rich systems show the lowest activity (Figure 9a). This finding is consistent with the results of Raman analysis, which suggested disorder in Co-rich structures with a tendency for Co to occupy specific positions in cationic sublattices, exposing Co-Co active sites at Se vacancy positions. The more Co-Co active sites present, the better the activity. Similarly to monometallic materials, there is an increase in the ECSA during measurement, suggesting a similar mechanism related to defect formation and Se particle detachment. However, the significantly higher ECSA and RF for the Co-rich sample compared to monometallic Co is intriguing (Figure 9b,c). This is likely related to the much higher amount of metal-like phases (judging by the intensity ratio in XRD) and the presence of the ordered cubic phase. The material with an equimolar cation proportion shows the highest activity among all studied samples, corresponding to the structural studies. Assuming an ordered structure and the dominance of cubic phases, one would expect the fewest defects and, thus, the lowest RF (Figure 9c). On the other hand, multimetallic active sites, evenly distributed within the structure, may lead to synergistic effects that enhance HER activity [33].

## 3. Experimental Section

### 3.1. Synthesis

**Scheme 1:**
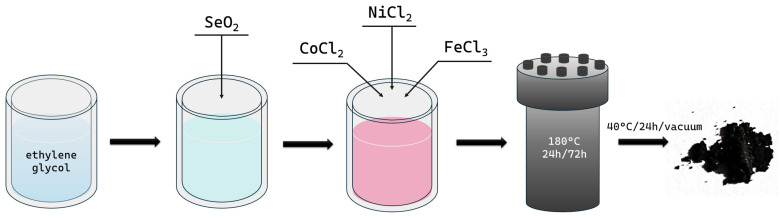
A schematic illustration of the selenide-based materials’ synthesis.

All reagents and chemicals were used as received without any further purification. Powders were prepared via solvothermal synthesis. Firstly, selenium dioxide (SeO_2_, Chemat, Gdańsk, Poland) was dissolved in 160 mL of ethylene glycol (EG, POCH, Gliwice, Poland). Afterwards, metal chlorides were added. Before the mixture was placed in the Teflon-lined stainless steel autoclave for 24 and 72 h at 180 °C, it was stirred for 15 min. The obtained materials were centrifuged, rinsed three times by ethanol (POCH, Gliwice, Poland), and dried at 40 °C under vacuum for 24 h. A scheme illustration of the synthesis is presented in Scheme 1. Detailed information about the chemical composition as well as the sample assays are listed in Table 3.

### 3.2. Materials Characterization

The samples’ morphological characterization was performed by scanning electron microscopy (SEM, ThermoFisher Scientific Apreo, Waltham, MA, USA).

The crystal structure of the obtained materials was investigated using an X’Pert MPD diffractometer (Malvern Panalytical Ltd., Malvern, UK). The system worked in the Bragg–Brentanno geometry. Phase identification was conducted using X’Pert HighScore Plus software (version 3.0.4) and the Powder Diffraction File (PDF2).

The structural properties of the samples were also examined by performing Raman imaging using the WITec Alpha 300 M+ spectrometer, equipped with a Zeiss 100× objective, 488 nm diode laser, and 600 gr/mm grating. Data processing was carried out using WITec ProjectFIVE Plus 5.3 software.

Electrodes were prepared using the following protocols: 5 mg of the obtained powders was sonicated in a solution of isopropyl alcohol (200 µL), distilled water (700 µL), and Nafion (100 µL). Afterward, 3 µL of the mixture was drop-cast on a clean carbon screen-printed electrode (Metrohm DropSens, Oviedo (Asturias) Spain) and left to dry in air at room temperature.

Cyclic (CV) and linear sweep (LSV) voltammetry techniques were applied to determine the electrochemically active surface area (EASA) of the electrodes and HER activity before and after stability tests, respectively. EASA measurements were recorded with 0.1 M KCl + 3 mM [Fe(CN)_6_]^3−/4−^ as an electrolyte at different scan rates (ν, 20–1000 mVs^−1^). For hydrogen evolution activity, electrodes were first conditioned by several LSV cycles to achieve steady-state conditions. All electrochemical measurements were conducted using an electrochemical analyzer Interface 1010 TM Potentiostat/Galvanostat/ZRA (Gamry Instruments, Warminster, PA, USA).

## 4. Conclusions

The research presented in this study investigated the structural and electrochemical characteristics of transition metal-based selenides in both binary and quaternary systems. Powders were synthesized using a solvothermal route. The presence of hexagonal selenium in all samples suggests incomplete reactions. The elongated reaction time affects the phase composition and crystallinity of the powders. Including additional d-block metals in quaternary systems increases configurational entropy, potentially leading to more homogenized and stabilized structures dominated by energetically favorable components, such as nickel. It was also proven that extending the synthesis time influences the stretching process. Electrochemical analysis reveals that binary systems exhibit fully reversible redox reactions, with nickel selenide-based samples exhibiting the highest electrochemically active surface area (EASA) of 6.6 mm^2^. Quaternary systems show varying electrochemical stability, with TF2 showing the most promising results. The assumption of equal contributions of nickel, cobalt, and iron appears beneficial in achieving chemically stable electrodes, though synthesis conditions require further optimization.

## Figures and Tables

**Table 1:** Methods for obtaining selected transition metal selenides.

System	Material	Synthesis	Application	References
binary	WSe_x_	hot-injection	hydrogen evolution catalysts	[25]
	Ni_3_Se_2_ FeSe	electrodeposition	oxygen evolution reaction	[26]
	Cu_2-x_Se	solid-state reaction	thermoelectric materials	[16]
	FeSe_2_	Schlenk line techniques	optoelectronic devices	[20]
	NiSe_2_	thermal selenization	hydrogen evolution reaction	[17]
ternary	WSe_x_, CoWSe_x_ FeWSe_x_ MnWSe_x_	hot-injection	hydrogen evolution catalysts	[25]
	(Ni_0.85_Fe_0.15_)_3_Se_4_ (Ni_0.24_Co_0.76_)Se (Co_0.1_Fe_0.9_)_3_Se_4_	electrodeposition	oxygen evolution reaction	[26]
quaternary	Co_3_Fe_3_Ni_3_Se_8_	solid-state reaction	hydrogen evolution reaction	[14]
	EuLnCuSe_2_	thermolysis	-	[27]
	Cs[RE_9_Mn_4_Se_18_] (RE = Ho–Lu)	solid-state reaction	-	[28]
	ACuMnSe_2_ (A = K, Rb, Cs)	thermal salt melting	-	[29]

**Table 2:** Characteristic parameters of the obtained modified electrodes.

Sample	EASA (mm^2^)	From HER Analysis			
		η_2_	η_10_	RF	RF after
F_Ni2	6.6	−593	-	7.43·10^−4^	1.37·10^−3^
F_Co2	2.5	−440	−601	4.42·10^−5^	2.93·10^−4^
F_Fe2	4.9	−572	-	5.03·10^−4^	1.20·10^−3^
TF2	4.4	−461	−620	4.42·10^−5^	8.32·10^−4^
TF_Ni2	3.1	−593	-	8.12·10^−4^	1.79·10^−3^
TF_Co2	3.6	−484	-	6.87·10^−4^	1.70·10^−3^
TF_Fe2	5.1	−493	-	5.83·10^−4^	1.54·10^−3^

**Table 3:** Synthesis parameters and sample assays.

Sample	Initial Chemical Composition					Reaction Time
	Se	Ni	Fe	Co	EG	
	mmol				mL	h
F_Ni	1	2	-	-	160	24
F_Fe		-	2	-		
F_Co		-	-	2		
F_Ni2	1	2	-	-	160	72
F_Fe2		-	2	-		
F_Co2		-	-	2		
TF	3	1	1	1	160	24
TF_Ni		2	1	1		
TF_Fe		1	2	1		
TF_Co		1	1	2		
TF2	3	1	1	1	160	72
TF_Ni2		2	1	1		
TF_Fe2		1	2	1		
TF_Co2		1	1	2		

## Data Availability

The dataset is available on request from the authors.
